# Supplementary material for: Causal interplay between lactose intolerance and gut microbiota: a combined bidirectional Mendelian randomization and in vivo validation study
Source: Front Nutr. 2026 Jun 1;13:1803337. doi: 10.3389/fnut.2026.1803337 (PMC13265576; doi:10.3389/fnut.2026.1803337)
Supplement: Supplementary file 1 [file Data_Sheet_1.zip › supplementary materials/Forward/leave one out cross validation/ebi-a-GCST90027588.finngen_R12_E4_LACTONAS.pdf]

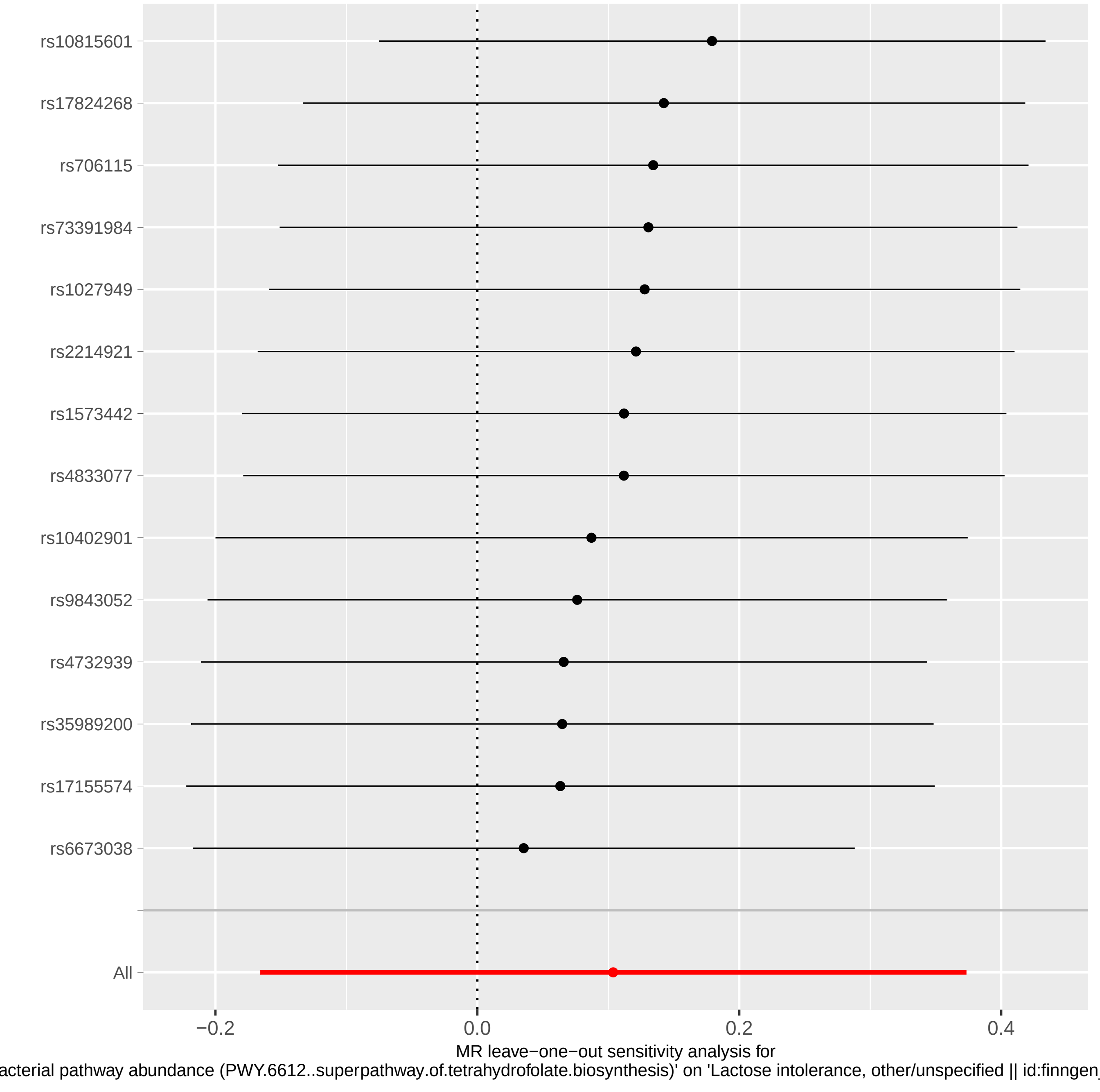
rs10815601
rs17824268
rs706115
rs73391984
rs1027949
rs2214921
rs1573442
rs4833077
rs10402901
rs9843052
rs4732939
rs35989200
rs17155574
rs6673038
All
−0.2
0.0
0.2
0.4
MR leave−one−out sensitivity analysis for
acterial pathway abundance (PWY.6612..superpathway.of.tetrahydrofolate.biosynthesis)' on 'Lactose intolerance, other/unspecified || id:finngen_
